# To feed or not to feed? Bioenergetic impacts of fear‐driven behaviors in lactating dolphins

**DOI:** 10.1002/ece3.3732

**Published:** 2017-12-27

**Authors:** Mridula Srinivasan, Todd M. Swannack, William E. Grant, Jolly Rajan, Bernd Würsig

**Affiliations:** ^1^ Office of Science and Technology National Marine Fisheries Service (NMFS) Silver Spring MD USA; ^2^ U.S. Army Engineer Research and Development Center Vicksburg MS USA; ^3^ Department of Biology Texas State University San Marcos TX USA; ^4^ Ecological Systems Laboratory, Department of Wildlife and Fisheries Sciences Texas A & M University College Station TX USA; ^5^ EcoLogik Consulting Group LLC Washington DC USA; ^6^ Department of Wildlife and Fisheries Sciences and Marine Biology Texas A & M University Galveston TX USA

**Keywords:** bioenergetics, lactation, marine mammals, predation risk effects, predator–prey interactions

## Abstract

In mammals, lactation can be the most energetically expensive part of the reproductive cycle. Thus, when energy needs are compromised due to predation risk, environmental disturbance, or resource scarcity, future reproductive success can be impacted. In marine and terrestrial environments, foraging behavior is inextricably linked to predation risk. But quantification of foraging energetics for lactating animals under predation risk is less understood. In this study, we used a spatially explicit individual‐based model to study how changes in physiology (lactating or not) and the environment (predation risk) affect optimal behavior in dolphins. Specifically, we predicted that an adult dolphin without calf would incur lower relative energetic costs compared to a lactating dolphin with calf regardless of predation risk severity, antipredator behavior, or prey quality consumed. Under this state‐dependent analysis of risk approach, we found predation risk to be a stronger driver in affecting total energetic costs (foraging plus locomotor costs) than food quality for both dolphin types. Further, contrary to our hypothesis, after accounting for raised energy demands, a lactating dolphin with calf does not necessarily have higher relative‐to‐baseline costs than a dolphin without calf. Our results indicate that both a lactating (with calf) and non‐lactating dolphin incur lowered energetic costs under a risk‐averse behavioral scheme, but consequently suffer from lost foraging calories. A lactating dolphin with calf could be particularly worse off in lost foraging calories under elevated predation risk, heightened vigilance, and increased hiding time relative to an adult dolphin without calf. Further, hiding time in refuge could be more consequential than detection distance for both dolphin types in estimated costs and losses incurred. In conclusion, our study found that reproductive status is an important consideration in analyzing risk effects in mammals, especially in animals with lengthy lactation periods and those exposed to both biological and nonbiological stressors.

## 1 INTRODUCTION

State‐based decision‐making under predation risk entails a trade‐off between feeding and vigilance (Brown & Kotler, 2004; Lima, 1998). A hungry animal may thus accept a higher level of risk compared to a satiated animal (Berger‐Tal, Mukherjee, Kotler, & Brown, 2010; McNamara & Houston, 1986; Olsson, Brown, & Henrik, 2002). Most animals tend to adopt a risk‐averse approach (McNamara & Houston, 1987), especially mothers with dependents. But risk‐averse behavior while foraging can have consequences in both time and energy lost (Lima, 1998). The energetic losses could be particularly severe for mammals with high reproductive costs during lactation (Lockyer, 1981; Young, 1976).

In mammals, lactation is the most energetically expensive part of the reproductive cycle (Lockyer, 2007), ranging from a two‐ to fivefold increase over base energy requirements (Clutton‐Brock, Iason, Albon, & Guinness, 1982; Gittleman & Thompson, 1988; Kastelein, Vaughan, Walton, & Wiepkema, 2002; Oftedal, 1985). Energy demands can vacillate during different lactation periods (early, mid or late) depending on species (Gittleman & Thompson, 1988). Also, lactation duration can last from a few weeks to multiple years for non‐human primates and marine mammals, with dolphins (*Odontocetes*) at the higher end of the scale (e.g., 36 months for *Tursiops truncatus*; Mann, Connor, Barre, & Heithaus, 2000). *Tursiops* sp. calves may also consume solid food between approximately 6 and 19 months (Kastelein, Staal, & Wiepkema, 2003), and continued suckling by calves may be to strengthen mother–calf social bonds.

Under state‐dependent risk theory (McNamara & Houston, 1987), we can expect that energy‐hungry females with young will evaluate both intrinsic (energy need) and extrinsic (resource availability and predation risk) factors to optimize decision‐making. Thus, mothers with offspring may choose to consume poor‐quality food to minimize predation risk, for example, bighorn sheep (*Ovis cadadensis*; Festa‐Bianchet, 1988) and red deer (Clutton‐Brock et al., 1982). Alternatively, mothers with young may tactically balance offspring survival and foraging requirements in high‐risk environments through habitat choice that facilitates escape or protection from predators while still maintaining nutritious food intake, as with bottlenose dolphins avoiding tiger sharks (*Galeocerdo cuvier*; Heithaus & Dill, 2002) and roe deer, (*Capreolus capreolus*) avoiding predation from red foxes (*Vulpes vulpes*; Panzacchi et al., 2010).

To compensate for the increased energy expenditure during lactation, mammals may use a combination of strategies. For example, migrating whales and most pinnipeds rely on metabolic stores during lactation (Costa, 2009; Miller, Best, Perryman, Baumgartner, & Moore, 2012). Smaller‐bodied species lack these metabolic stores and therefore are unable to fast for extended periods (Costa, 2009; Mann, 2009; Oftedal, 1997). They therefore reduce activity, which can serve as an ancillary strategy to overcome energy deficits, as in non‐human primates (Barrett, Halliday, & Henzi, 2006; Dufour & Sauther, 2002). Alternatively, animals may consume more food items or choose higher quality prey (Bernard & Hohn, 1989; Dias, Rangel‐Negrin, & Canales‐Espinosa, 2011; Malinowski, 2011).

We know from other studies that lactating animals manipulate their time/energy budgets to address potential energy deficits by resting and socializing less, while increasing vigilance (Barrett et al., 2006; Dunbar & Dunbar, 1988). For example, Laurenson (1995) found that in cheetahs (*Acionynx jubatus*), mothers with cubs in lair or emerging cubs had a higher food intake rate than solitary cheetahs, and compensated by a preference for larger‐sized prey during lactation. Also, cheetahs with cubs in lair rested less and traveled farther to hunt and seek water. Likewise, Dias et al. (2011) found that lactating black howler monkeys (*Alouatta pigra*) consumed more nutritious fruits and reduced activity to accommodate elevated energy demands, with variability between early and late lactation stages. To gauge the adaptability and variability among lactating mammals, we need to quantify elevated energy costs for reproductive female mammals due to changes in activity and foraging behavior normally and when under risk.

Furthermore, given that predator–prey interactions are not static (Lima, 2002; Sih, 1984), it is important to integrate the behaviorally dynamic interaction between predator and prey. When prey and predator are mobile and behaviorally adaptive, there are manifold end result permutations depending on whether predator or prey dominates. Therefore, calculation of bioenergetics under risk should ideally integrate the behavioral flexibility of predator and prey.

**Figure 1:**
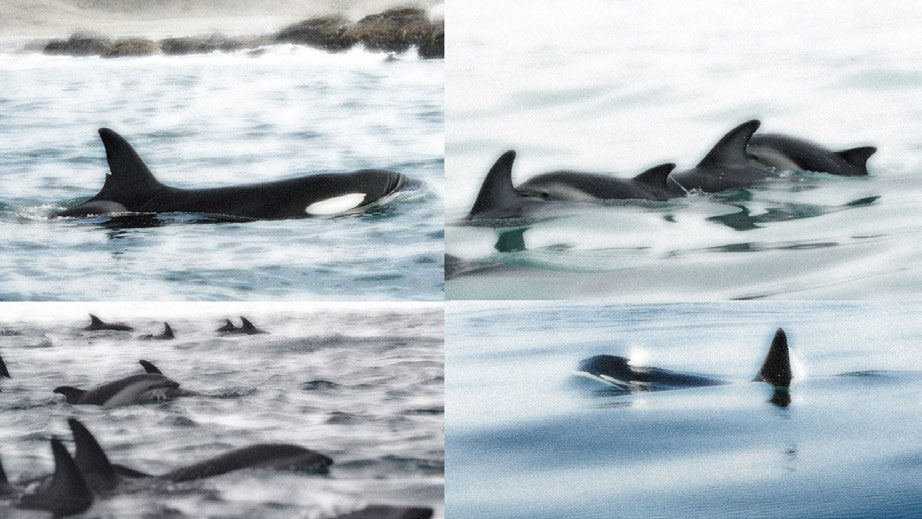
killer whales and dusky dolphins off Kaikoura, New Zealand

Within the operating paradigm described above and to address limitations in previous studies, we used a novel approach to quantify and compare relative energetic costs and losses experienced by a lactating versus non‐lactating dusky dolphin (*Lagenorhynchus obscurus*) under predation risk (Figure 1). We calculated the relative variability in energetic costs for a lactating adult (hereafter LD) and nonlactating adult (hereafter AD) as a function of multiple variables: high and low predation risk, high‐ and low‐quality prey, different prey capture costs, and risk‐averse to risk‐prone antipredator behavior. Accounting for higher baseline costs, we hypothesized that LDs should have higher total energetic costs and lost foraging calories than ADs regardless of food quality and severity of predation risk because of their risk‐averse decision‐making.

### 1.1 System of interest

**Figure 2:**
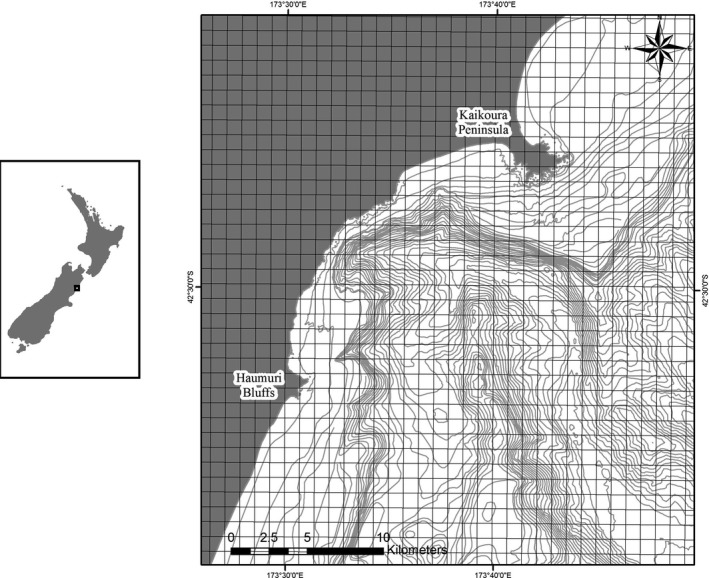
(Left) Map showing region of interest, Kaikoura, New Zealand. (Right) Enlarged map of the system of interest near Kaikoura, New Zealand indicating grid of 1,468, 1 km × 1 km cells used to represent the habitat of the model (land cells were excluded)

Dusky dolphins or “duskies” are an abundant southern hemispheric species, frequently encountered off Kaikoura, New Zealand (reviewed in Würsig, Duprey, & Weir, 2007), with the overall population near Kaikoura estimated at more than 12,000 animals, although no more than about 2,000 animals are there at any one time (Markowitz, 2004). The Kaikoura Canyon, New Zealand (42°30′S 173°35′E, Figure 2), is a deep‐sea U‐shaped submarine canyon that originates about 500 m from shore; it is roughly 60 km long and 1,200 m deep and is a prominent feature in the area. The canyon is marked by a subtropical convergence zone that supports a thriving population of marine animals (Lewis & Barnes, 1999). The Kaikoura Canyon is considered to be one of the most productive “biomass hotspots” in the deep sea (De Leo, Smith, Rowden, Bowden, & Clark, 2010) and is a prime foraging habitat for duskies.

The small (<2 m) and gregarious duskies rely on complex social networking to feed, mate, reproduce, and avoid predators (Würsig & Würsig, 2010). Based on long‐term (opportunistic) datasets and field observations since the 1980s, duskies show remarkable and strong avoidance response to the presence of killer whales (*Orcinus orca*) in the area (Dahood et al., 2008; Markowitz, 2004; Srinivasan & Markowitz, 2010; Weir, 2007; Würsig & Würsig, 1980). Direct observations of predation events are rare (Constantine, Visser, Buurman, Buurman, & McFadden, 1998; Visser, 1999), but there are multiple accounts of duskies fleeing at top speed when killer whales are detected, leaving the area entirely until the threat has subsided, or seeking refuge in shallow waters <10 m deep (*reviewed in* Srinivasan & Markowitz, 2010). In extreme situations, duskies may strand in <1 m deep waters (Cipriano, 1992). New Zealand killer whale–dusky dolphin interactions are both predatory and nonpredatory (*reviewed in* Srinivasan & Markowitz, 2010). Mixed dusky dolphin responses could reflect predator intent, predators losing the element of surprise, or prey preference—not all New Zealand killer whales have a preference for duskies (Visser, 1999).

Animal use of space demonstrates the trade‐off between foraging and risk minimization (Lima, 1998). Duskies likewise make strategic habitat choices that indicate risk‐averse behavior. For instance, duskies prefer near‐shore, shallow waters (<200 m) during peak killer whale presence between late austral spring until autumn (Dahood et al., 2008; Srinivasan & Markowitz, 2010; Weir, 2007) and during calving periods (Weir, Duprey, & Würsig, 2008). Mothers with calves break off of the main groups and take the added precaution of being in waters <20 m deep to possibly prevent incidental killer whale or shark attacks in deep waters and to avoid conspecific male harassment (Weir et al., 2008). When risk from killer whales is low in austral winter, duskies are found in much larger groups in deeper waters (>200 m; Markowitz, 2004).

The duskies’ chief food supply comes from feeding on an ascending layer of mesopelagic organisms at night (Würsig, Würsig, & Cipriano, 1989). These mesopelagic organisms are mostly comprised of myctophids (family Myctophidae), hoki (*Macruronus novaezelandiae*), and several species of squid (Cipriano, 1992). For a majority of the year, duskies of mixed age and sex classes make daily offshore trips to feed on mesopelagic organisms at night and then return to near‐shore waters to rest and socialize. Mothers with calves may also engage in daytime feeding to offset the inability to feed at night or to supplement nighttime consumption of mesopelagic fish and squid (Weir, 2007). Daytime feeding is rare (Cipriano, 1992; Markowitz, 2004). Lactation duration is estimated at approximately 18 months for New Zealand duskies (Leatherwood & Reeves, 1983) and about 12 months for duskies in Peru (van Waerebeek & Read, 1994). We assume that like other species, lactating duskies off Kaikoura optimize their time/energy budgets, allowing them to maximize foraging chances while minimizing predation risk.

## 2 METHODS

We modified an existing individual‐based model (IBM) to quantify the impacts of dynamic predator and prey interactions on the energetics of lactating prey species. IBMs are well‐established tools to explore ecological interactions that are difficult, if not impossible, to observe in nature (DeAngelis & Grimm, 2014). Specifically, we added a bioenergetics module to the model developed by Srinivasan, Grant, Swannack, and Rajan (2010), which was created to explore the evolution of antipredator behavior in dusky dolphins in the Kaikoura Canyon.

The IBM is a spatially explicit, grid‐based, geo‐referenced, stochastic IBM (Railsback & Grimm, 2012), programmed in VB.NET^©^ (Microsoft, 2003 and updated in VB 10 2010). The model contains three main components: habitat (which contains dusky dolphin prey resources), dusky dolphins, and killer whales. The habitat is based on geo‐referenced shapefiles from the Kaikoura Canyon region; behavioral rules and physical capacities determine the simulated movement of individual dusky dolphins and killer whales. We here briefly describe the model components pertinent to this study, emphasizing the behavioral rules and the new bioenergetics module. We direct the readers to Srinivasan et al. (2010) for the complete model description and evaluation (the parameterization and functions remain the same as Srinivasan et al., 2010, with the exception of the addition of lactating females and a bioenergetics module). Our experimental design for this study explores varying combinations of the distance at which prey can detect predators (and vice‐versa), predation intensity, predation avoidance, and dusky dolphin prey food quality.

### 2.1 Habitat

The habitat within the model is represented by geo‐referenced bathymetry contours obtained from shapefiles in and around the Kaikoura Canyon, New Zealand. There are 1,468 one km^2^ grid cells in the model domain (Figure 2), which was defined based on dolphin surveys (Cipriano, 1992; Markowitz, 2004) and sightings recorded from tour boats (Dahood et al., 2008). Each grid cell contains a depth (calculated as average depth between two bathymetric contour lines in a cell) and a prey for the dusky dolphins (described below). Cell size was determined based on swimming speeds of dusky dolphins and killer whales such that simulated animals moving at maximum velocity can only move to an adjacent cell in one time step, which was 1/16 hr. The time period for simulations was a 210‐day period beginning one hour before sunset on 1 November, based on observed killer whale presence in the system.

### 2.2 Dusky dolphins

Dusky dolphin behaviors can be divided into six behavioral states (i) *rest,* (ii) *travel,* (iii) *search,* (iv) *feed,* (v) *flee,* and (vi) *hide*. *Rest* for mothers and calves tends to be during the day in shallow waters (near the coastline), *travel* is to deeper water to *search* for food and then *feed* at night, then *travel* back to shallower water once the duskies have met their daily energetic requirements. For our purposes, we assume exclusive nighttime feeding in offshore waters.

A core feature of the IBM is the capture of the behavioral dynamics between predator and prey, which affects outcome and prey decision‐making (Srinivasan et al., 2010). Duskies will follow normal habitat use and movement rules until they detect killer whales, at which point they respond by *fleeing* and *hiding* in shallow waters <10 m deep for a prescribed amount of time, depending on the scenario being tested.

We did not differentiate between velocities in different behavioral states for the LD and AD. In reality, a mother with a calf in her slip stream may experience more drag when swimming (Noren, 2008). However, Weihs (2004) showed that dolphins with calves are capable of maintaining similar flight speeds as adult dolphins without a calf. In the model, the only parameters different for the LD were the longer hiding times (1, 3, and 12 hr) postkiller whale encounter compared to adult dolphin hiding times (0.25, 1, and 9 hr). We assumed that an LD would be more cautious and remain in a refuge well after the threat had dissipated and/or would potentially leave the area upon detection of killer whales (Srinivasan & Markowitz, 2010). For example, duskies on occasions have been observed to stay in shallow refuges for ~4 hr (Cipriano, 1992), but the timing could vary based on threat levels (Lima, 1998). The other parameters remained unchanged from Srinivasan et al. (2010).

### 2.3 Dusky dolphin energetics

For foraging energetics calculations, we assumed that the mesopelagic layer is available only at night for dolphins to feed on. Based on acoustic studies off Kaikoura and Hawaii (Benoit‐Bird, 2004; Benoit‐Bird, Dahood, & Würsig, 2009), distribution of the deep scattering layer‐associated organisms appears uniform near Kaikoura. Although numerical prey density is less than near Hawaii, exact numbers are unavailable for Kaikoura. The amount of food available per day and prey density is unknown. Acoustic studies on the deep scattering layer off the Hawaiian Islands show that the mean caloric density of the mesopelagic boundary (island‐associated) community is 83 kcal/m^3^; with a maximum of 9,000 kcal/cu m (Benoit‐Bird, 2004). Therefore, we assigned an index of 0–100 kcal/cu m of food per cell with water depths > 400 m (Reid, 1994). We do not consider lunar effects (Benoit‐Bird et al., 2009), which may affect dolphin foraging behavior and decisions.

Daily energetic requirements (DER) for duskies to meet their maintenance needs were assumed at 50 kcal kg^−1^ day^−1^ for a 70 kg adult, roughly 3,505 kcal/day (Cipriano, 1992). We assumed a DER of 3,500 kcal/day for all of our calculations, but did not differentiate energy requirements into separate growth, reproductive, or thermoregulatory costs.

Lactating dolphins are expected to have higher energetic needs relative to other adult dolphins. For example, energy needs for lactating captive bottlenose dolphins were between 1.5 and 3 times higher than baseline levels (Kastelein et al., 2002; Reddy, Kamolnick, Curry, Skaar, & Ridgway, 1994). Model‐based estimates for energy requirements for a lactating Pacific white‐sided dolphin (*Lagenorhynchus obliquidens*) were about 1.4 times higher than total energy requirements for adults in MJ/day (Rechsteiner, Rosen, & Trites, 2013). For model purposes, we assumed doubling of DER for the LD, amounting to 7,000 kcal/day.

Although data are sparse, dusky dolphin individual and group foraging behavior in Kaikoura likely varies based on prey density and distribution within the water column (Benoit‐Bird, Würsig, & McFadden, 2004; Benoit‐Bird et al., 2009).

Dolphins maintain a search area of 5 sq. km to find a suitable “food” cell with water depths > 400 m. One female 70 kg dusky dolphin in captivity consumed roughly 10% of her body weight (Kastelein, van der Elst, Tennant, & Wiepkema, 2000). In contrast, a 78 kg Pacific white‐sided dolphin was estimated to consume between 16% and 20% of its body weight (Rechsteiner et al., 2013). Food consumption is likely to be higher in the wild, but food intake per unit of body weight is generally smaller with increasing animal size (Kleiber, 1975). In the model, we assume that dolphins feed throughout the period the mesopelagic layer is available, although feeding may be highest when dolphin prey rises closest to the surface around midnight (Benoit‐Bird et al., 2004). Dolphins are assumed to continue feeding interspersed with food searching or other activity, for example, when interrupted by a predator.

Food is assumed plentiful and no restrictions are placed on foraging patch availability and density, or vertical and horizontal migration rates. Within the model, given the parameters discussed above, we recorded time spent foraging/feeding and distance covered in travel and flee mode.

### 2.4 Dolphin foraging energetics calculations

We calculated two metrics to characterize dusky dolphin energy expenditure for the five scenarios described above: (i) total energy expenditure and (ii) foraging calories lost.

### 2.5 Total energy expenditure

We calculated Dusky Total Energy Expenditure (kcal/day) = Foraging Costs (kcal/day) + Locomotor Costs (kcal/day), where locomotor costs = Mean Distance Travel + Mean Distance Flee

In the equation, locomotor costs for travel includes distances traveled by LD and AD on a daily basis and locomotor costs for fleeing (if applicable) for the five model treatment scenarios.

#### 2.5.1 Estimating foraging costs (FC)

Foraging Cost = 0.15 kcal fixed search cost per prey item + capture costs (expressed as 5%, 10%, 15%, and 20% of caloric content of prey)

[Based on Benoit‐Bird, 2004, for Hawaiian spinner dolphins (*Stenella longirostris*)*,* prey energy value is derived from dusky dolphin stomach content data].

Similar to Benoit‐Bird (2004), we assume duskies preferentially seek larger prey and “search costs” include searching, diving, and feeding on prey and is independent of prey size. A 0.15 kcal fixed search cost is a low estimate and corresponds to about an 8% increase over the animal's maintenance energy needs during active foraging (Benoit‐Bird, 2004).

### 2.6 Dusky stomach content data

To obtain caloric content of prey and relative energy contribution to the duskies’ overall DER, we used dusky dolphin stomach content data published in Cipriano (1992). Cipriano provided a summary of stomach contents for duskies obtained from strandings, incidental and opportunistic captures for all seasons—summer, fall, spring, and winter (*n* = 26). He used 13 dusky dolphin specimens to obtain estimates of prey energetic content for the three most common prey items identified above, based on literature values over the prey length range consumed by duskies.

For purposes of foraging cost calculations, we used only six of 13 dolphin specimens, including only dolphins with full guts and disregarding outliers in terms of weight or length. The dolphin specimens considered for analysis weighed between 69 and 77 kg with a total length 160–186 cm and were composed of fresh, intact prey parts (Appendix S2). As the exact prey size/length from stomach contents is not known, to calculate net value of prey, we used both the high and low estimate for total prey energy value (based on prey size) percentage contribution to DER of the dolphin (3,500 kcal; Appendix S2). The average of percentage prey energy contribution was used to determine how much more dolphins must eat to meet a DER of 3,500 and 7,000 kcal/day (depending on dolphin type; see Appendix S3).

### 2.7 Foraging calories lost

Foraging calories lost by a dolphin when exposed to varying levels of predation risk were calculated based on LD and AD feeding rates of 330 and 675 kcal/hr, respectively, assuming a DER of 7,000 and 3,500 kcal/day, and foraging throughout duration of food availability, which in turn is tied to diel cycles. Specifically,

$$\text{Foraging calories lost}=\frac{{\text{DER}}_{\mathrm{dusky}}}{\text{foraging time}}\times \left(\text{Time flee}+\text{Time Hide}\right)$$

where foraging time depends on the amount of time dolphins spent feeding and searching for food derived from the model simulation results and Time flee + Time Hide.

#### 2.7.1 Estimating locomotor costs (LC) under predation risk

Cost of Transport (COT) is typically defined as the metabolic costs of moving one unit of mass one unit of distance (Schmidt‐Nielsen, 1972). For calculating LC, we used the Rosen and Trites (2002) allometric equation LC = 1.651M^1.01^, where M is mass in kg and the absolute energy costs are measured in joules/m. The exponent in the equation suggests that cost of swimming is proportional to body mass = 70 kg for an adult dolphin. The equation is suitable for determining locomotor costs in bioenergetic models for animals with limited empirical data (Rosen & Trites, 2002). For consistency, all units were converted into kcal/day in the calculations and LC (Distance Travel + Distance Flee) was calculated for all five scenarios.

### 2.8 Killer whale (predator)

Killer whales enter the system day and night and thus are capable of hunting during the day or night (Deecke, Shapiro, & Miller, 2013; Newman & Springer, 2007). There is an equal probability that killer whales will enter the system from the north or south side and take a shallow or deep water bathymetric contour track (see Srinivasan et al., 2010 for additional details). Temporal and spatial variations in killer whale density or predation risk are achieved through their probabilistic entry into the system and through establishment of six killer whale return rates from four times per day (highest risk) to once every 10 days (lowest risk).

Once killer whales enter the system, they have four behavioral states: (i) *cruise/search,* (ii) *stalk,* (iii) *wait, and* (iv) *posthunt*. Killer whales *cruise/search* along the bathymetric contour line they chose until they detect a dolphin at which point they *stalk* (i.e., chase) the dolphin. If the killer whale catches the dolphin, it enters the *posthunt* state. If the dolphin escapes to shallow water (<10 m), the killer whale will wait for a prescribed amount of time, then enter a *posthunt* state if the dolphin does not reemerge. During the *posthunt* state, killer whales do not attack but stay in the vicinity and presumably are either feeding or seeking another opportunity to hunt.

### 2.9 Experimental design

Prey avoidance by dolphin instance and killer whale predator strategies is, in principle, a derivative of varying prey detection distance (low to high) and hiding times (low to high). These antipredator maneuvers reflect conditions where prey has been ineffective in assessing predator threat and motivation, or in other words, predators were able to successfully avoid detection and increase their hunting chances. To capture the spectrum of possible reactions to varying predation risk, we developed five treatment scenarios, including a baseline, which also provide the basis for calculating energy budgets for the dolphins.

The scenarios range across the *fear driven* (safe strategy) to the *fear impulse* (risky strategy) and are defined as:

**Table:** 

Baseline Strategy: Background strategy that Kaikoura duskies are assumed to adopt in response to intermittent killer whale threats. This strategy is represented by optimal prey detection distances and hiding times
scenario 2: Fear‐driven Strategy: This strategy minimized encounters with killer whales (long hiding time after encounter and large detection distances)
scenario 3: Fear Impulse Strategy: This strategy maximized feeding time (short hiding time after encounter and short detection distances)
scenario 4: Maximized Detection/Minimum Hiding Time or Max. Detection/Min. Hiding: This strategy maximized detection distance while minimizing time spent in the refuge by the duskies
scenario 5: Maximized Hiding Time/Minimized Detection or Max. Hiding/Min. Detection: This strategy maximized time spent in the refuge by the duskies

We calculated caloric costs of nightly foraging behavior (foraging costs, locomotor costs, and foraging calories lost) for both LD and AD, under each of these five scenarios, under each of six predation risk levels (killer whale return intervals of 0.25, 0.5, 1, 3, 5, and 10 days), and under each of four dolphin prey capture costs represented as a percentage caloric content of prey item consumed (5%, 10%, 15%, and 20%).

For each of the five scenarios, we ran 20, one‐year, Monte Carlo (replicate stochastic) simulations with LD and AD for a total of 200 simulations. We recorded mean foraging time and mean number of killer whale (KW) encounters in addition to time/distance budgets for the different behavioral states for each set of simulations.

To illustrate the trade‐off between predation risk and food quality on energetic consequences for a LD and AD, we chose a subset of the parameters: low (10 days KW return rate) and high (four times/day KW return rate) predation risk, low and high food quality at a fixed prey capture cost of 15% on relative energetic costs and foraging calories lost for the different scenarios constructed. Complete results for all multivariate combinations are available in the Supporting Information (Appendix S4).

We used R system for statistical computing (R Core Team 2014) to conduct all statistical tests. As data were both non‐normal and heteroscedastic, and transforming “*y*” variables did not alter assumptions, we first conducted one‐way ANOVA tests to determine treatment (scenario) differences for both LD and AD. We then conducted an additional Welch's two sample *t* test to distinguish any statistically significant differences in behavior and foraging energetic costs for LD and AD based on different prey capture costs and prey quality and across all scenarios.

## 3 RESULTS

Overall, there were statistically significant differences between treatments (scenarios) for the time/distance behavioral variables tested for AD and LD (Appendix S5a). We also found statistically significant differences in time/distance behavioral variables used in foraging energetics calculations and number of killer whale encounters between LD and AD across all scenarios except for proportion of time spent fleeing and hiding (Appendix S5b).

**Figure 3:**
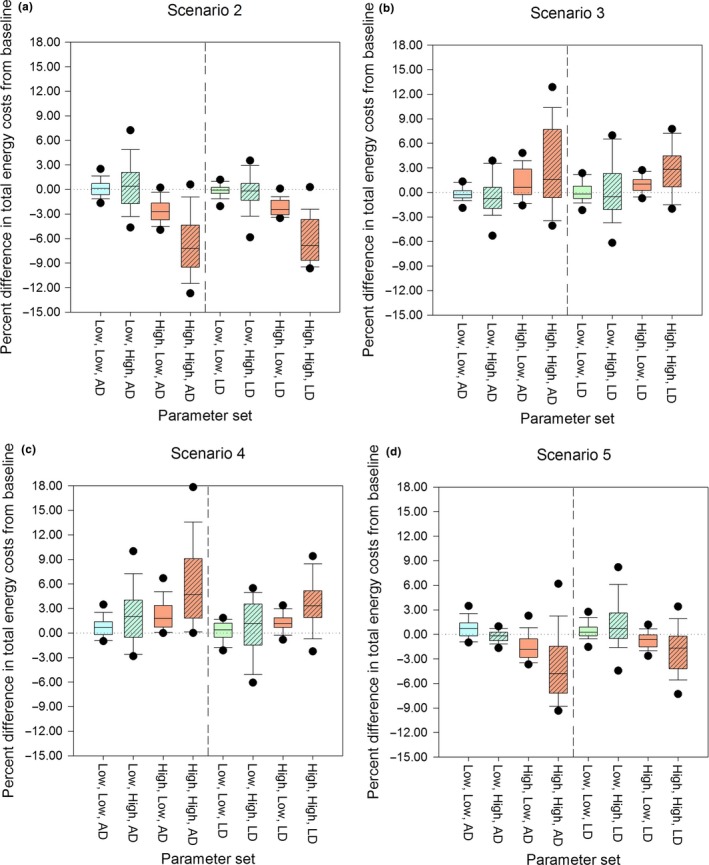
Total energy expenditure (foraging costs + cost of transport) for LD and AD in reference to respective baseline energy costs. The first parameter is predation risk and the second is food quality and are investigated at two levels: low and high

**Figure 4:**
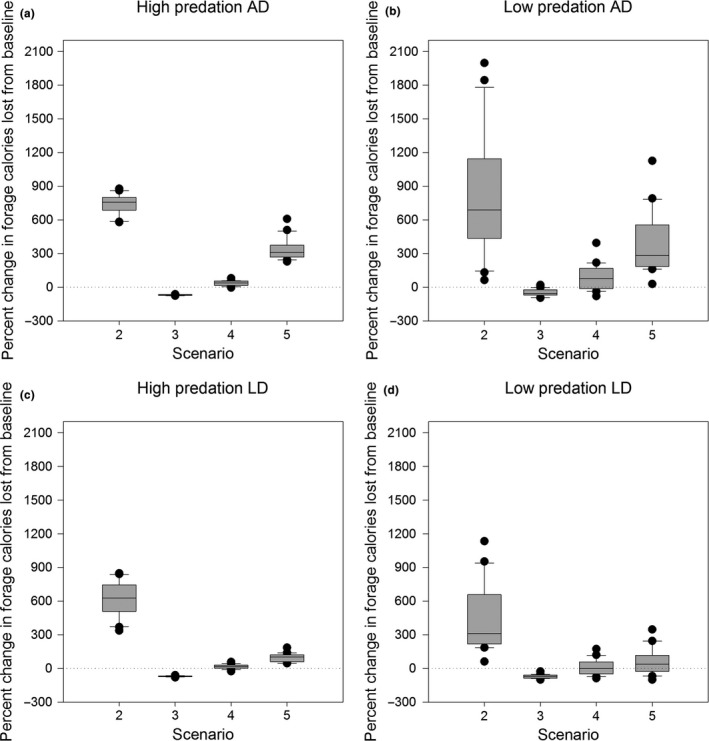
Relative change in estimated foraging calories lost (FCL) from baseline for LD and AD under high and low predation risk

The next set of figures (Figures 3 and 4) elucidate the interplay between food quality and predation risk for an LD and AD under specified scenarios and relative to the baseline (assumed background strategy for dusky dolphins near Kaikoura).

Figure 3 represents relative total energy costs under low and high risk, conditional upon quality of food consumed and antipredator decision‐making. Specifically, Figure 3 shows the percentage change from the baseline total energy costs (Foraging + Locomotor Costs) for LD and AD (*y*‐axis) under low/high predation risk and low/high food quality for each scenario (2, 3, 4, and 5). The left half of the figure corresponds to AD and the right to LD.

In scenario 2 (Figure 3a), both LD and AD experience median costs similar to the baseline for low predation risk/low food quality with more variability seen in AD. There was a minimal increase (~1%) for AD and slight decrease for LD (<1%) in median costs under low predation risk/high food quality, but there was more variability for AD. Under/high predation risk/low food quality, both LD and AD have similar median costs, an ~3% decline from the baseline. For high predation risk/high food quality, LD and AD exhibit a near 7% decline in median costs with AD showing a maximum decrease of ~9% and LD a little less than that. However, despite similar overall trends, LD shows less variability in energy costs relative to AD.

In scenario 3 (Figure 3b), there is limited difference relative to baseline (scenario 3) under low food quality/low predation risk for both LD and AD. Under low predation risk/high food quality, both LD and AD experience a median cost of roughly 1% less than baseline with more variability in LD than AD energy costs. Under high predation risk/low food quality, LD experiences a higher (~2%) median cost than AD (1%) relative to the baseline, but AD can experience a maximum increase in energy costs of ~3%. When predation risk and food quality are high, both AD and LD experience higher energy costs. However, AD has a lower median cost than LD, but has a maximum energy cost of ~8% higher than baseline levels, compared to 5% for LD.

In scenario 4 (Figure 3c), under low food quality/low predation risk, there is a minor increase in median energy expenditure for both LD and AD. The costs increase for both LD and AD under low predation risk/high food quality with AD experiencing a slightly higher median cost than LD. These costs remain largely unchanged under high predation risk/low food quality for both groups although there is less variability than in the previous category. Under high predation risk/high food quality, we see both LD and AD experience higher median costs relative to baseline, but AD could experience elevated median costs of 5% compared to ~3% for LD with a maximum increase of 9%, whereas LD maximum costs are estimated to be ~5%.

In scenario 5 (Figure 3d), median costs are similar to scenario 4 under low predation risk/low food quality for both LD and AD and are ~1% higher than baseline. The median costs increase for LD under low predation risk/high predation risk by ~2%, but do not deviate remarkably from baseline for AD. Under high predation risk/low food quality, AD experience a drop of ~2% in median costs, whereas decrease is minimal for LD. Finally, under high predation risk/high food quality, AD median costs are ~5% lower than baseline compared to about a 2% median decrease for LD. There is also considerable variability in energy expenditure for AD compared to LD. Also, AD could experience a maximum decrease in costs of ~8%.

Overall, for both LD and AD, the greatest decrease from baseline occurs under scenario 2 followed by scenario 5 (Min. Det./Max. Hiding) and under high predation risk/high food quality. Conversely, greatest increases are observed under scenarios 3 and 4 and again with maximum change observed under high predation risk/high food quality. In general, LD costs are lower than comparable AD levels and show less variability in the range of estimated energy costs relative to baseline under most parameter combinations tested.

A result of lost feeding time and energy due to antipredator maneuvers is reflected in shifts in foraging calories lost (FCL) from baseline for LD and AD under predation risk (Figure 4).

Overall and contrary to our hypothesis, AD surprisingly experiences a higher FCL shift from baseline than LD across all scenarios and variation in predation risk. When predation risk is high, the FCL values do not drastically depart from baseline for LD except in scenario 2 (Figure 4c) and under scenarios 2 and 5 for AD (Figure 4a). Similar to AD, the baseline equivalent or fewer losses are observed under scenario 3. Median losses range from about 600 kcal/day in scenario 2 to ~100 kcal/day under scenario 5 for LD (Figure 4c) whereas for AD median losses incurred are estimated to range from ~800 kcal/day in scenario 2 to ~300 kcal/day in scenario 5 (Figure 4a). Under low predation risk (Figure 4b), AD experiences the maximum FCL under scenarios 2 and 5, ranging from a median loss of ~700 kcal/day in scenario 2 to ~300 kcal/day relative to the baseline. Fewest losses occur in scenario 3 followed by scenario 4. For LD (Figure 4d), the trends are similar to AD, but median losses are comparatively lower and range from ~300 kcal/day in scenario 2 to ~100 kcal/day in scenario 5.

## 4 DISCUSSION

Lactating mothers are attractive targets for predators due to the presence of calf and the reduced ability of the mother–young pair to escape (Barrett et al., 2006; Caro, 1987). Female mammals with *k‐selected traits* make huge energy investments in reproduction and parental care to ensure survival of offspring (most marine mammals produce a single offspring every 1–3 years; Berta, Sumich, & Kovacs, 2006). For some marine mammals, for example, sea otters, lactation can be a significant physiological drain on energetic reserves (Thometz, Kendall, Richter, & Williams, 2016). The energetic deficits could potentially be intensified by additional man‐made (ocean noise) or natural stressors (e.g., predators). Therefore, there is tremendous incentive for marine mammals to optimize decision‐making in lifestyle choices.

At the beginning of this paper, we hypothesized that LD would have higher foraging costs and more lost foraging calories than AD regardless of food quality and severity of predation risk because of their generally risk‐averse decision‐making and doubled DER. To test this, we explored the impacts on relative energetic (largely foraging) costs in comparison with individual LD and AD baseline (our assumed background strategy for duskies in Kaikoura) and by concentrating on the high and low ends of the predation risk spectrum and fixed prey capture costs (15%) for consumption of high and low nutrition prey.

Our results reveal foremost that for both LD and AD, (i) risk‐averse behavior (scenario 2 and scenario 5) is energetically costly and (ii) high predation risk levels have greater impact than food quality. This is particularly evident when we compare low predation risk/high food quality and high predation risk/high food quality. Predictably, longer hiding times (scenarios 2 and 5) result in decreased energy costs, particularly when predation risk is higher. Overall, AD experience a higher net energetic “cost” relative to baseline level than LD depending on the situation. For both LD and AD, there is a greater departure from baseline levels under high food quality/high predation risk with median costs declining under scenarios 2 and 5, and costs increasing in scenarios 3 (short detection range and hiding times) and 4 (large detection range). Thus, impulsive decisions under risk yield little energetic rewards and conversely, an overtly conservative tactic may prove energetically expensive.

When we distinguish by reproductive status, we surmise that the greater variability and higher costs for AD relative to baseline could be due to the less cautious approach adopted by AD versus LD. The more conservative habitat choices and risk‐averse strategic behaviors do not cause distinct swings in energetic costs as scenarios change. The importance of strategic risk‐averse behavior for LD is particularly evident in scenario 3, where LD experience higher relative costs than AD when both detection ranges and hiding times are curtailed. Further, in our behavioral rules, we stipulated a longer hiding window in refuge for LD compared to an AD postencounter with a predator, whereas detection distances for both LD and AD would be the same (Table 1). Based on our results in scenario 5, there may be an optimum hiding interval for LD given much sharper decreases in foraging costs for AD under the same scenario relative to baseline.

**Table 1:** List of treatments to compare variation in prey–predator behavior dynamics and calculate bioenergetics for a lactating dolphin with calf (LD) and adult dolphin without calf (AD)

No.	scenario^a^	AD	AD	LD	LD
		Detection distance (km)	Hiding time (hr)	Detection distance (km)	Hiding time (hr)
1	Baseline strategy	5	1	5	3
2	Fear‐driven strategy	10	9	10	12
3	Fear impulse strategy	1	0.25	1	1
4	Maximum detection/minimum hiding time	10	0.25	10	1
5	Minimum detection/maximum hiding time	1	9	1	9

a For each scenario, we simulated six different killer whale presences: appearing 1, 2, 4 times per day and every 3, 5, or 10 days. For representing results, we used a low predation risk = 10 days and high predation risk = 0.25 days.

Thus, contrary to our hypothesis, LD with elevated DER do not necessarily have higher relative foraging costs than AD. Furthermore, all parameters being equal between LD and AD, the reduced variability in overall energetic costs for LD suggests a more optimal decision‐making scheme when exposed to risk. In other words, their choice of shallower water depth and closer proximity to refuge may give them an advantage over AD without significantly compromising foraging costs.

Nonetheless, lowered foraging costs may not be an energetically beneficial outcome. We know that time spent vigilant, whether through habitat selection or hiding and fleeing instead of foraging, translates into foraging calories lost (Dill & Fraser, 1997). Thus, an assessment of differences in FCL between LD and AD (Figure 4) risk‐averse behavior leads to increased FCL for both LD and AD. But in the case of AD, predation risk does not seem to alter median FCL. For LD, high predation risk has a doubling effect on FCL only under scenario 2 with higher detection distance and hiding time. There appears to be limited impact on FCL under scenario 5 despite longer hiding times for LD and AD. Losses are least when risk taking is highest in scenarios 3 and 4. Time spent in refuge appears to have a stronger influence than detection range on both foraging costs incurred and FCL. However, the effects visibly shift when both are maximized or minimized. Undoubtedly, these effects are amplified under heightened risk.

These results provide some supporting evidence for the evolutionary practicality of large lactation costs and lengthy investment in calf rearing among dolphins and primates. These results are not atypical and have been modeled in other studies (McNamara & Houston, 1994), but provide fresh perspectives on possible optimal solutions for lactating animals attempting to control predation rates while maintaining energetic needs.

When resources are abundant, animals can sometimes exhibit prolonged avoidance of predators but show increased acceptance of predation risk when resources are limited (Dill & Gillett, 1991). Animals can also vary hiding time based on food availability (Dill & Fraser, 1997; e.g., *Serpula vermicularis* tube‐dwelling polychaete worms). As increase in risk taking is proportional to increase in short‐term food availability (Abrams, 1991), when food is predictable, hiding and re‐emergence times are not likely to be an energetic burden. In the dusky dolphin system, food is predictable even if restricted in its availability, but delayed emergence from refuge could prove energetically burdensome over time.

Fortunately for duskies, proximity to food and likely food abundance ensures that duskies, regardless of social strata, are not energetically deprived unless food fluctuates and becomes increasingly difficult to access due to environmental change or prey shifts. With fluctuating risk‐averse behaviors and raised energetic demands, lactating dolphins cannot afford to miss feeding opportunities. Supplementary daytime feeding may be important for mothers with calves to compensate for cautious behavior and to replenish depleted energy reserves. This is likely true for adults as well.

A secondary approach to reducing energy deficits is to invest in nutrient‐rich prey. Previous studies have suggested that food *quality* could be sometimes more significant than food *quantity* in driving marine predator population dynamics and foraging behavior (Österblom, Olsson, Blenckner, & Furness, 2008; Spitz et al., 2012). Based on stomach content analysis, Bernard and Hohn (1989) documented that lactating spotted dolphins (*Stenella attentuata*) may choose poor‐quality food to stay with their infants at the surface longer, while nonlactating females consumed more high‐energy prey. If food is abundant and risk is low, a dusky dolphin mother could be very selective about her prey choice (Malinowski, 2011). Thus, there is a penalty for a mother with dependent young, and accordingly, she has to supplement feeding, increase food quantity, or be nutritively selective until physiologically constrained (Lockyer, 1993; Perez & Mooney, 1986). Prey choice may also be dictated by lactation stage (early, mid, late), as variations in milk composition have been noted in both dolphins and whales (West et al., 2007). Under the scenarios we tested and at the two extreme predation risk levels, we found that while high food quality reduces foraging costs, heightened predation risk or disturbance can obviate the nutritional benefit offered by being prey selective.

Another potential way of managing energy needs is by reducing activity levels while still being vigilant through habitat selection and behavior (Dias et al., 2011; Laurenson, 1994). From previous studies (Markowitz, 2004; Weir, 2007), we know that the predominant daytime behavioral state for mothers with calves is rest. Our model simulation also confirmed these observations (Srinivasan et al., 2010). Similar to other species (Barrett et al., 2006), for duskies, reduced activity can be assumed to be a preferred lactation strategy to manage elevated energy demands and maintain heightened vigilance (early detection of risk).

**Figure 5:**
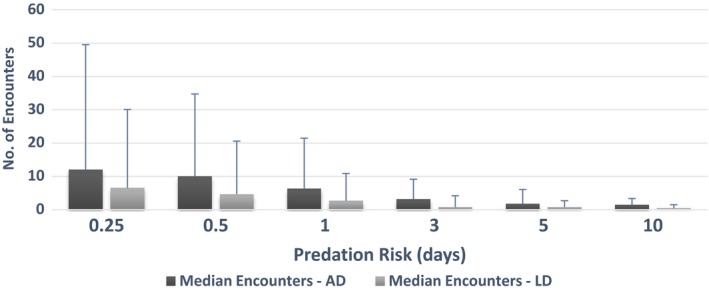
Differences between LD and AD in estimated median number of killer whale‐dusky dolphin encounters across all five model scenarios as a function of decreasing predation risk (0.25–10 days; *x*‐axis). LD, lactating dolphin; AD, adult dolphin (no calf). Error bars indicate standard deviation

Dusky dolphins socially segregate in their habitat selection and thus differ in “costs” associated with these behaviors. The AD with deeper daytime depth preferences travels less than the LD with shallow water preference. This does not compound travel costs for either dolphin as Kaikoura Canyon's topography facilitates close access to food‐rich deep waters. In the model, we did not differentiate between flee and travel velocities for the LD, so there could be a significant cost attached to echelon swimming, significantly reducing maternal speed (Noren, 2008; Figure 5).

Risk‐averse strategic choices have clear implications for animal survival. As most killer whales and sharks have been observed in water depths >20 m (Markowitz, 2004; Weir, 2007), a clear benefit of the depth choice is that dusky dolphin mothers dragging infants in their slip stream have to cover much shorter distances to reach shallow water refuge (<10 m deep waters) compared to AD. Further, across all scenarios considered, LD have an estimated median encounter rate of ~7 (min = 1.2, max = 49; scenario 3) compared to 12 (min = 3.6, max = 80; scenario 3) for the AD under the highest predation risk (Figure 4). Thus, maternal choice is in part supported by reduced estimated encounter rates with killer whales, as well as through photographic evidence of limited to no scarring (Srinivasan, 2009).

To quantify energetic costs under risk, we estimated both foraging and locomotor costs. As discussed above, locomotor costs appear insignificant for these dolphins as part of their background strategy and under different predation risk scenarios. Recently, Williams et al. (2017) predicted an increase of ~30% in metabolic rate for a beaked whale in flight (continuous stroking combined with reduced glide time) after exposure to ocean noise. So in the future, we may need to consider flight costs and their impact on total energetic expenditure for dolphins with and without calf.

In our study, the greatest contribution to overall energetic costs is from foraging costs or cost of predation risk (Brown & Kotler, 2004). However, we believe our calculated foraging costs and consequently overall energy estimates are conservative, as they do not explicitly account for (i) seasonal food availability, (ii) variable food patch density, (iii) depletion of patch by thousands of feeding dolphins, (iv) dolphin time/depth constraints on feeding, and (v) variation in individual satiation and hunger levels.

Food availability and food intake rate, while directly linked, are also affected by forager motivational state (hungry vs. satiated forager; Caraco, 1980; Charnov, 1976). Although within the model design we prevent the dolphins from feeding ad libitum by forcing them to move to a different cell if a food patch is depleted to two‐third of its original density (giving up density), and food availability is restricted to cells >400 m deep, the latter does not prevent food acquisition as the Kaikoura canyon bathymetry ensures easy access to deep waters. The travel distance to a “foraging cell” is estimated to be short for duskies and therefore contributes to minimal travel and food searching costs. This could change as food distribution changes or is available much farther from shore. Our limited knowledge about the foraging behavior of duskies off Kaikoura and of characteristics of the food density and distribution keeps us from a more sensitive measurement of foraging cost and success and remains an important gap to address in future studies. Improving our knowledge of the fluctuating predation pressure from large sharks and killer whales is another area for further study to assess impacts on day and nighttime foraging behaviors.

Lactation is a feature unique to mammalian reproduction with complex selective pressures operating at the family unit level (Dall & Boyd, 2004; Hayssen, 1993). Evolutionarily, lactation across different mammalian species is in a constant mode of flux as energy needs must be synchronized with resource availability, and these needs consequently influence reproductive timing, as well as other behavior and physiological attributes (Eisert & Oftedal, 2009; Hayssen, 1993; Pond, 1977). A key determinant of reproductive success is body condition, which is dependent on foraging behavior (Hamel & Côté, 2009). Females with poor body condition can sometimes fail to reproduce in subsequent years as observed in herbivorous mammals (Clutton‐Brock et al., 1982; Hamel & Côté, 2009). Typically, lactating mammals with their increased energy demands and lactation strategies can suffer from poor body condition if they cannot compensate for energy requirements (Miller et al., 2012), and a weak animal can be a particularly easy target for inefficient predators (Wirsing, Steury, & Murray, 2002). Furthermore, there is strong evidence that maternal condition influences offspring phenotype (Rossiter, 1996), and sustained exposure to disturbance or predators could potentially lead to prolonged stress with possible reproductive failure (Clinchy, Zanette, Boonstra, Wingfield, & Smith, 2013; Sheriff, Krebs, & Boonstra, 2010).

Frid and Dill (2002) likened risk effects to acoustic disturbance effects, with similar population‐level consequences. Off Kaikoura, recent evidence suggests that tour boats have a chronic impact on duskies similar to predator effects (Lundquist, 2012). We also know that environmental disturbance (e.g., El Niño) can influence maternal body condition and pregnancy rates (Ono, Boness, & Oftedal, 1987; Williams et al., 2013). We speculate that the frequency, duration, and intensity of the disturbance will have greater fitness costs for a lactating dolphin than those in other social classes, particularly in environments where they are exposed to aggregated impacts from human activities and predation risk. Thus, we need to be cognizant of potential energy tipping points for a lactating mother relative to nonreproductive adults when assessing population‐level impacts from disturbance or other sublethal stressors.

## CONFLICT OF INTEREST

None declared.

## AUTHOR CONTRIBUTIONS

MS framed the research question and conducted model simulations. MS and TS developed the study design, refined the model, and analyzed results. WG, JR, TS, and MS were involved in the model design, development, and testing. BW provided critical expertise in study improvements and overall project guidance. All authors were involved in manuscript preparation and writing.
