# Supplementary material for: To feed or not to feed? Bioenergetic impacts of fear‐driven behaviors in lactating dolphins
Source: Ecol Evol. 2017 Dec 27;8(2):1384–98. doi: 10.1002/ece3.3732 (PMC5773337; doi:10.1002/ece3.3732)
Supplement: Supplementary file 1 [file ECE3-8-1384-s001.docx]

**Supplementary Information**

**Appendix 1.** List of class attributes and aggregated variables of the IBM, including units of measure, baseline values outside parenthesis, and information sources. Unless specified, the same parameters and values were used for a lactating mother with calf and an adult dolphin without a calf.

| **Class Attributes** | **Values** |
| --- | --- |
| **(b) Dolphin class** | (With Calf, WC= 1; No Calf, NC = 0) |
| Location | X-Y coordinates |
| Behavioral state | Rest, Travel, Feed, Flee, or Hide |
| Distance to detect killer whales (km) | WC & NC = 5 (1, 5, 10) |
| Hiding time (hr)^a^ | WC = 3 (1, 3, 12) NC = 1 (0.25, 1, 9 ) |
| Resting velocity (km hr^-1^)^a, b^ | 4 |
| Foraging velocity (km hr^-1^) | 5 |
| Traveling velocity (km hr^-1^)^b^ | 8 |
| Fleeing velocity (km hr^-1^)^b,^ | 18 |
| **(c) Killer Whale class** |  |
| Location | X-Y coordinates |
| Behavioral state | Cruise, Stalk, Wait, or Post-hunt |
| Distance to detect dolphins (km) | 5 |
| Reentry Interval (days)^d, e^ | 3 (0.25 – High Risk, 0.5, 1, 3, 5, 10 – Low Risk ) |
| Post-hunt time (hr) | 1 |
| Waiting time (hr) | 1 |
| Average Cruising velocity (km hr^-1^)^f,g^ | 8 |
| Average Stalking velocity (km hr^-1^)^f-h^ | 16 |
|  |  |
| **(a) Habitat class** |  |
| Spatial location | X-Y coordinates |
| Depth | m |
| DSL | Yes or No^*^ |
| **(d) Aggregated variables** |  |
| Total Distance | Km |
| Distance Travel | Km |
| Distance Rest | Km |
| Distance Forage | Km |
| Distance Flee | Km |
| Time Rest | Proportion |
| Time Travel | Proportion |
| Time Hide | Proportion |
| Time Flee | Proportion |
| Time Forage | Proportion |
| Total Killer Whale Days in System | Number |
| Predator-prey Encounters | Number |
| ^a^ Ciprano (1992); 1 to 4 hr |  |
| ^b^ Markowitz (2004); between 16 and 22 km hr^-1^ |  |
| ^c^ Dahood et al. (2008) |  |
| ^d^ Srinivasan and Markowitz (2009) |  |
| ^e^ Dolphin Encounter tour boat data (1995 - 2007) |  |
| ^f^ Williams (2002); 12.6 (cruising) – 43 (sprinting speed) km hr^1^ |  |
| ^g^ Ford et al. (2005); 15 to 30 km hr^1^ (Stalking/chasing velocity) |  |
| ^h^ Ford and Reeves (2008); >15 to 20 km hr^1^ (Stalking/Chasing Velocity) |  |
| ^*^stochastic variable |  |

**Appendix 2:** Summary of stomach content of dusky dolphins (n = 6) with fresh prey used to determine prey energy content and dusky dolphin foraging costs with and without calf. Squid species included: *Nototodoras sp., Moroteuthopsis sp., Teuthowenia sp.,* and unidentified squids. Percentage contribution of prey meal to dusky dolphin daily energetic needs were based on power output/energetic efficiency estimates of Hui 1987. All stomach content data and energy values are based on Cipriano (1992).

| **Specimen No.** | **Dusky Length (cm)** | **Dusky Weight  (kg)** | **Gut state** | **Prey (Fresh and intact prey)** | |  |  | **Estimated  Energy Value (kj)** | **Estimated %  of dusky dolphin daily energy requirements (^##^Low to High)** |
| --- | --- | --- | --- | --- | --- | --- | --- | --- | --- |
|  |  |  |  | **^#^Squid** | **^#^Myctophids** | | **^#^Hoki** |  |  |
| 85FC02 | 186 | 77 | Full | 3 |  | | 7 | 14750 | 29.9-100.4 |
| 85FC03 | 180 | 75 | Full | 3 |  | | 1 | 2900 | 5.9-19.7 |
| 86FC01 | 175 | 77.5 | Full | 1 |  | | 5 | 10250 | 20.8-69.8 |
| 87FC01 | 175 | 77 | Full | 4 | 206 | | 2 | 11580 | 23.5-78.8 |
| 88FC02 | 167 | 69 | Full | 10 | 2 | | 4 | 12060 | 24.4-82.1 |
| 88FC03 | 160 | 71 | Full | 5 |  | | 1 | 3650 | 7.4-24.9 |
| # Refers to number of prey items represented by intact squid, squid head, intact fish or Hoki skulls | | | | | | | | | |
| ## Low and High Energy values used to calculate foraging costs for dolphins | | | | | | | | | |

**Appendix 3.** Summary of Foraging Costs estimated for AD and LD based on Daily Energetic Requirements (DER) of the 2 dolphin types and 3 different prey capture costs. Locomotor Costs (LC) estimated for AD and LD when killer whales are not present in the system and is the reference value for LC calculations (see Figure 2) under variable predation risk and model treatment scenarios 1-5. LD = Lactating Dolphin; AD = Adult Dolphin (without calf)

| ****FORAGING COSTS (FC)** |  |  |  |  |  |
| --- | --- | --- | --- | --- | --- |
| **Prey capture costs** | **5%** | **10%** | **15%** | **20%** |  |
| Average # of prey items based on dusky dolphin full gut contents ( n = 6 dolphin specimens) | 42.3 |  |  |  |  |
| Fixed search cost | 0.15 |  |  |  |  |
| **LOW PREY ENERGY VALUE CONTRIBUTION TO DAILY ENERGETIC REQUIREMENT (DER)** |  |  |  |  |  |
| Dolphins have to feed x times to be full based on LOW percentage prey energy value contribution to DER, where x = | 5.36 |  |  |  |  |
| Total estimated foraging cost for AD | 622.8052 | 1211.6012 | 1800.397 | 2389.193 | kcal/day |
| Total estimated foraging cost for LD with a DER of 7,000 kcal/day | 1245.6104 | 2423.2024 | 3600.794 | 4778.386 | kcal/day |
|  |  |  |  |  |  |
| **HIGH PREY ENERGY VALUE CONTRIBUTION TO DAILY ENERGETIC REQUIREMENT (DER)** |  |  |  |  |  |
| Dolphins have to feed x times to be full based on high percentage prey energy value of DER of 3,500 kcal/day, where x = | 1.6 |  |  |  |  |
| Total estimated foraging cost for AD | 185.912 | 361.672 | 537.432 | 713.192 | kcal/day |
| Total estimated foraging cost for LD with a DER of 7,000 kcal/day | 371.824 | 723.344 | 1074.864 | 1426.384 | kcal/day |
|  |  |  |  |  |  |
| **LOCOMOTOR COSTS (LC) ( KILLER WHALES ABSENT, REFERENCE)** |  |  |  |  |  |
| AD (Distance Travel) | 125.409 | kcal/day |  |  |  |
| LD (Distance Travel) | 257.418 | kcal/day |  |  |  |
| ****Calculations based on Cipriano (1992) and Benoit-Bird (2004)** |  |  |  |  |  |
| **Foraging cost = 0.15 Kcal fixed search cost per prey + capture cost expressed as a percentage caloric content of prey** |  |  |  |  |  |

**Appendix 5**

**a) One-way ANOVA results to distinguish treatment differences (Scenarios 1-5) for both AD and LD (AD = Adult dolphin with no calf; LD = Lactating dolphin with calf), b) Welch’s two sample t-test results to distinguish differences in time/distance behavioral variables tested for LD and AD, c) Welch’s two sample t-test results comparing total energy costs at different prey capture costs and consumption of high and low calorie prey for LD and AD.**

| ***Distance Travel*** |
| --- |
| AD: F = 39.5848, num df = 4, denom df = 290.778,p < 0.01 |
| LD: F = 36.6438, num df = 4, denom df = 297.069,p< 0.01 |
|  |
| ***Distance Flee*** |
| AD: F = 6.8815, num df = 4, denom df = 295.704, p < 0.01 |
| LD: F = 5.4896, num df = 4, denom df = 295.832,p< 0.01 |
|  |
| ***Distance Total*** |
| AD:F = 62.9156, num df = 4, denom df = 283.689,p< 0.01 |
| LD:F = 96.3448, num df = 4, denom df = 288.402,p< 0.01 |
|  |
| ***Number of Encounters*** |
| AD:F = 84.1367, num df = 4, denom df = 275.946, p< 0.01 |
| LD:F = 73.8154, num df = 4, denom df = 276.262, p < 0.01 |
|  |
| ***Proportion Time Feed*** |
| AD:F = 60.3845, num df = 4, denom df = 278.089,p< 0.01 |
| LD:F = 54.6332, num df = 4, denom df = 277.915,p< 0.01 |
|  |
| ***Proportion Time Flee*** |
| AD:F = 62.4069, num df = 4, denom df = 276.704,p< 0.01 |
| LD:F = 73.1848, num df = 4, denom df = 275.039,p< 0.01 |
|  |
| ***Proportion Time Hide*** |
| AD:F = 99.2313, num df = 4, denom df = 269.689, p< 0.01 |
| LD:F = 78.1697, num df = 4, denom df = 270.828,p< 0.01 |
|  |
| ***Foraging Calories Lost*** |
| AD:F = 87.111, num df = 4, denom df = 254.003,p< 0.01 |
| LD:F = 73.7062, num df = 4, denom df = 255.256,p< 0.01 |

**b)**

| ***Distance Travel (LD vs. AD)*** |
| --- |
| t = -70.1389, df = 910.434, p<0.01,95% CI -913.995 -864.238 |
|  |
| ***Distance Flee (LD vs. AD)*** |
| t = 11.3479, df = 1039.223, p<0.01,95% CI 108.039 153.215 |
|  |
| ***Distance Total (LD vs. AD)*** |
| t = -14.4024, df = 1194.094, p<0.01, 95% CI -1195.592 -908.908 |
|  |
| ***Number of Encounters (LD vs. AD)*** |
| t = 6.1639, df = 1001.845, p<0.01, 95% CI 4.308 8.332 |
|  |
| ***Proportion Time Feed (LD vs. AD)*** |
| t = 6.2444, df = 1176.491, p<0.01, 95% CI 0.010 0.0186 |
|  |
| ***Proportion Time Flee (LD vs. AD)*** |
| t = 0.067, df = 1186.731, p = 0.9466, 95% CI -0.0020 0.0022 |
|  |
| ***Proportion Time Hide (LD vs. AD)*** |
| t = -2.3191, df = 1171.793, p = 0.021, 95% CI -0.018 -0.002 |
|  |
| ***Foraging Calories Lost (LD vs. AD)*** |
| t = -7.8956, df = 747.035, p<0.01, 95% CI -1134.138 -682.461 |

**c)**

| ***5% Capture Costs HighAD and HighLD*** |
| --- |
| t = -27.356, df = 55.391, p<0.01, 95% CI -311.253 -268.768 |
|  |
| ***10% Capture Costs HighAD and HighLD*** |
| t = -43.935, df = 55.391, p<0.01, 95% CI -487.013 -444.528 |
|  |
| ***15% Capture Costs HighAD and HighLD*** |
| t = -60.5141, df = 55.391, p<0.01, 95% CI -662.772 -620.288 |
|  |
| ***20% Capture Costs HighAD and HighLD*** |
| t = -77.0931, df = 55.391, p<0.01, 95% CI -838.532 -796.048 |
|  |
| ***5 % Capture Costs LowAD and LowLD*** |
| t = -68.5672, df = 55.391, p<0.01, 95% CI -748.146 -705.661 |
| ***10% Capture Costs LowAD and LowLD*** |
| t = -124.1071, df = 55.391, p<0.01, 95% CI -1336.942 -1294.457 |
|  |
| ***15% Capture Costs LowAD and LowLD*** |
| t = -179.6469, df = 55.391, p<0.01, 95% CI -1925.738 -1883.253 |
|  |
| ***20% Capture Costs LowAD and LowLD*** |
| t = -235.1868, df = 55.391, p<0.01, 95% CI -2514.534 -2472.049 |
